# Experiences, perceptions and utilization of CareLine: an evaluation study

**DOI:** 10.3389/fpubh.2025.1667535

**Published:** 2025-12-11

**Authors:** Jane Mingjie Lim, Pearline Lee, Daniel Sng, Felicia Kua, Choon How How, Hong Choon Oh

**Affiliations:** Changi General Hospital, Singapore, Singapore

**Keywords:** social isolation, tele-befriending, older adults, mixed methods, program evaluation

## Abstract

**Background:**

Social isolation and loneliness significantly impact the physical and mental health of older adults. To address these issues, telephone-based care services such as CareLine, a 24/7 service in Singapore for seniors living alone, provide emotional support, social interaction, and emergency response. This study evaluates the utilization and experiences of CareLine users and providers to understand how effectively the service meets the needs of older adults.

**Methods:**

A mixed methods approach was used, combining a retrospective analysis of CareLine’s call log dataset (November 2016 to January 2024) and in-depth interviews with users and staff. Quantitative data was analyzed using descriptive and multivariable regression analyses to explore participant characteristics and engagement patterns. Qualitative data from 20 interviews were thematically analyzed to explore user and provider perspectives.

**Results:**

The call log data included 9,512 users, with 318,950 recorded calls over the study period. Most users were female (64.6%), Chinese (82.1%), and aged 80 years or older (33.2%). Call volume peaked during the COVID-19 pandemic, with social care and emotional support being the most common call topics. Male users, those not currently married, and individuals living alone were more likely to engage with the service. Frequent callers (1% of the users) contributed to 68.5% of the inbound call volume. Thematic analysis highlighted the value of cultural and linguistic compatibility in service delivery, but also pointed to operational challenges, particularly with staffing and call management.

**Conclusion:**

CareLine effectively provides social and emotional support to a vulnerable population of older adults, particularly those living alone. However, frequent callers and high call volumes during the pandemic have stretched the service’s capacity, highlighting the need for targeted user engagement strategies, increased staffing, and resource optimization. The findings have important implications for the design and implementation of telephone-based care services for older adults globally.

## Introduction

Social isolation and loneliness are pervasive issues globally, with evidence indicating that up to one-third of older adults in some countries experience feelings of loneliness (1). Prior research has also documented the association between loneliness and poor physical and mental health (2), as well as the effect of social isolation on older adults’ overall quality of life (3) – the impact of these factors on mortality is comparable to other recognized risk factors such as smoking, obesity, and lack of physical activity (4–6).

To alleviate the lack of regular social interactions among older adults, befriending services which pair older adults with volunteers or paid workers for regular social interaction and emotional support, have emerged as a promising intervention. These interactions can occur face-to-face, over the telephone, or through other communication mediums, aiming to reduce loneliness, enhance psychological well-being, and improve overall quality of life. Numerous studies have evaluated the effectiveness of befriending services for older adults. In particular, a study evaluating a helpline service targeting loneliness among older adults in the UK found that while the service attracted individuals who needed frequent emotional support, it successfully fostered satisfying relationships among distinct individuals (7). Other case studies conducted to in Northern Ireland found that key mechanisms such as reciprocity, empathy, autonomy and privacy were important in building relationships with individuals and contributed to the effectiveness of these interventions.

In Singapore, where a rapidly aging population is occurring alongside increasing numbers of older adults living alone and evolving familial caregiving norms, there remains limited research on telephone-based befriending services. Given this context, this paper aims to explore the implementation and impact of CareLine, a telephone-based care service established in 2016 as a 24/7 caLL center run by Changi General Hospital. The intervention was conceptualized as a multi-pronged approach to build a foundational relationship with older adults over 60 years who lived alone, provide emergency response to those in distress, as well as to facilitate regular social interaction and emotional support through telephone communication. CareLine also ensures that older adults are connected to appropriate community resources, particularly in cases where they may be unaware of whom to contact for assistance. Since its inception, CareLine has expanded nationwide and is now accessible to all seniors, regardless of living arrangement, with over 20,000 currently enrolled in the program.

Despite the global evidence base on tele-befriending services, important gaps remain. Existing evaluations have been concentrated in Western settings (8–10), with limited attention to real-world implementation in multicultural Asian contexts. Furthermore, few studies adopt a mixed-methods approach that captures both utilization patterns and lived experiences over an extended period (11). While quantitative analyses of service utilization can provide insights into patterns of engagement, call frequency, and demographic differences, these data alone cannot explain the nuanced motivations, barriers, or perceived value of the service from the perspective of older adults and providers. Qualitative data is then needed to capture lived experiences, relational dynamics, and contextual factors shaping service effectiveness in a multicultural Asian setting (12, 13). By integrating both forms of evidence, the evaluation offers a holistic understanding of how tele-befriending services function in practice, informs culturally sensitive adaptations, and generates lessons for scaling and sustainability.

This study aims to examine the utilization and experience of both CareLine users and providers in understanding current call characteristics and how effectively older adults’ needs are being met. This will also contribute to the growing body of global evidence on the effectiveness of telephone-based befriending interventions and offer insights into best practices and efficient resources for interventions related to the well-being of older adults living alone.

## Methods

We conducted a mixed methods study to understand current utilization patterns of CareLine, as well as both user and provider perspectives of the intervention. First, we performed a retrospective analysis using the CareLine call log dataset to examine utilization patterns of the service. Subsequently, we conducted in-depth interviews with both CareLine users and providers to gather qualitative insights into their experiences and perspectives. Topic guides used can be found in Appendix A.

The call log dataset consisted of all calls made to and received by CareLine from November 2016 to January 2024. All call records were included in data analysis. Available variables included anonymised participant demographics, living arrangements, year of enrolment and call-related information such as date, time, call type (inbound or outbound), and call outcome (successful or unsuccessful). Call types and outcomes were coded by the CareLine call agent who attended to the call.

**Figure 1:**
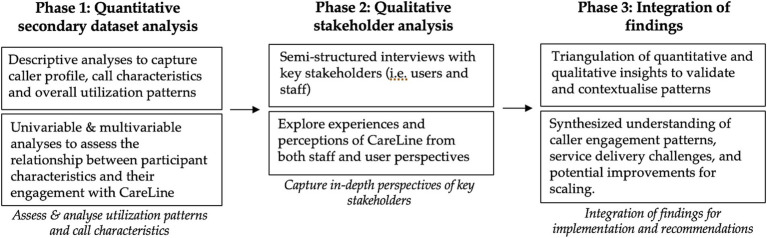
Mixed methods research framework.

The study was guided by an adapted mixed methods research framework for healthcare process improvement (14) (Figure 1).

### Recruitment and data collection

Descriptive and univariable analyses were used to describe caller profile, call characteristics, and overall utilization patterns. We then conducted multivariable logistic regressions to understand the relationship between participant characteristics and their engagement with CareLine.

We informed and triangulated these findings with qualitative data from 20 key informant in-depth interviews, including both CareLine staff and users. Participants were eligible if they were over 21 years old, able to understand and provide consent, and had either been enrolled in or working with CareLine for at least 12 months. CareLine users were purposively sampled based on call frequency to ensure representation of both high- and low-frequency users, with recruitment continuing until thematic saturation was reached. Staff participants were selected to capture diverse perspectives across roles, including operations, calling agents, as well as leadership positions. Those who were willing to participate were interviewed either over the phone or face-to-face by a member of the research team (JL, JW). Interviews were conducted in both English and Chinese by a member of the research team. All interviews were audio recorded without personal identifiers. The interviews followed a semi-structured approach and interview guides were used for all participants. Interview guides were developed through previous literature and key topics were further contextualized and distilled through discussions with the CareLine team.

During the interview, CareLine staff were asked about their experiences with working with CareLine and the existing capacity, resources and partnerships that facilitate the delivery and scaling of services. With seniors, questions were related to their perceptions of CareLine, as well as their general experiences with loneliness.

Interviews with participants ranged between 20 to 45 min each, and all audio recordings of the interviews were transcribed verbatim and translated into English when needed. Transcriptions were stripped of any identifiers and imported into Dedoose (15) to facilitate data coding, retrieval and analysis. The data was analyzed thematically using an inductive (data driven) approach, and coding was conducted independently by two members of the research team (JL, JW). For the purposes of this analysis, we coded according to the key topics described above, with discrepancies resolved by consensus. Intercoder reliability was assessed during the initial coding phase to ensure consistency in code application. Participant quotations are provided below to illustrate our findings.

Ethics approval for this study was exempted by the Centralized Institutional Review Board (CIRB).

## Results

### Call characteristics and volume

The retrospective call log dataset contained a total of 9,512 participants were enrolled in the CareLine program from November 2016 to January 2024. Prior to 2020, participants were commonly recruited through physical roadshows and door-to-door outreach efforts. However, due to COVID-19 social distancing measures, recruitment of CareLine users subsequently took place over the phone. The majority of users were female (64.6%) and Chinese (82.1%), with a significant portion of participants aged 80 years and above (33.2%) and living alone (44.0%). More participant characteristics can be found in Table 1.

**Table 1:** Participant characteristics.

CareLine users/frequentcallers	CareLine users (*n* = 9,512)	Frequent callers (*n* = 64)
Gender, *n* (%)		
Female	6,143 (64.6)	34 (53.1)
Male	3,369 (35.4)	30 (46.9)
Age, *n* (%)		
<65 years	2,318 (24.4)	3 (4.7)
65–69 years	816 (8.6)	10 (15.6)
70–74 years	1,454 (15.3)	13 (20.3)
75–79 years	1766 (18.6)	21 (32.8)
80 + years	3,158 (33.2)	17 (26.6)
Ethnicity, *n* (%)		
Chinese	7,811 (82.1)	46 (71.9)
Malay	563 (5.9)	12 (18.8)
Indian	994 (10.4)	6 (9.3)
Others	144 (1.5)	
Marital, *n* (%)		
Widowed	2,973 (31.3)	18 (28.1)
Married	2,938 (30.9)	18 (28.1)
Single	2,565 (27.0)	24 (37.5)
Divorced	898 (9.4)	4 (6.3)
Separated	101 (1.1)	0 (0)
Not reported	37 (0.4)	0 (0)
Living, *n* (%)		
Living with others	3,551 (56.0)	29 (45.3)
Living alone	4,181 (44.0)	35 (54.7)

**Figure 2:**
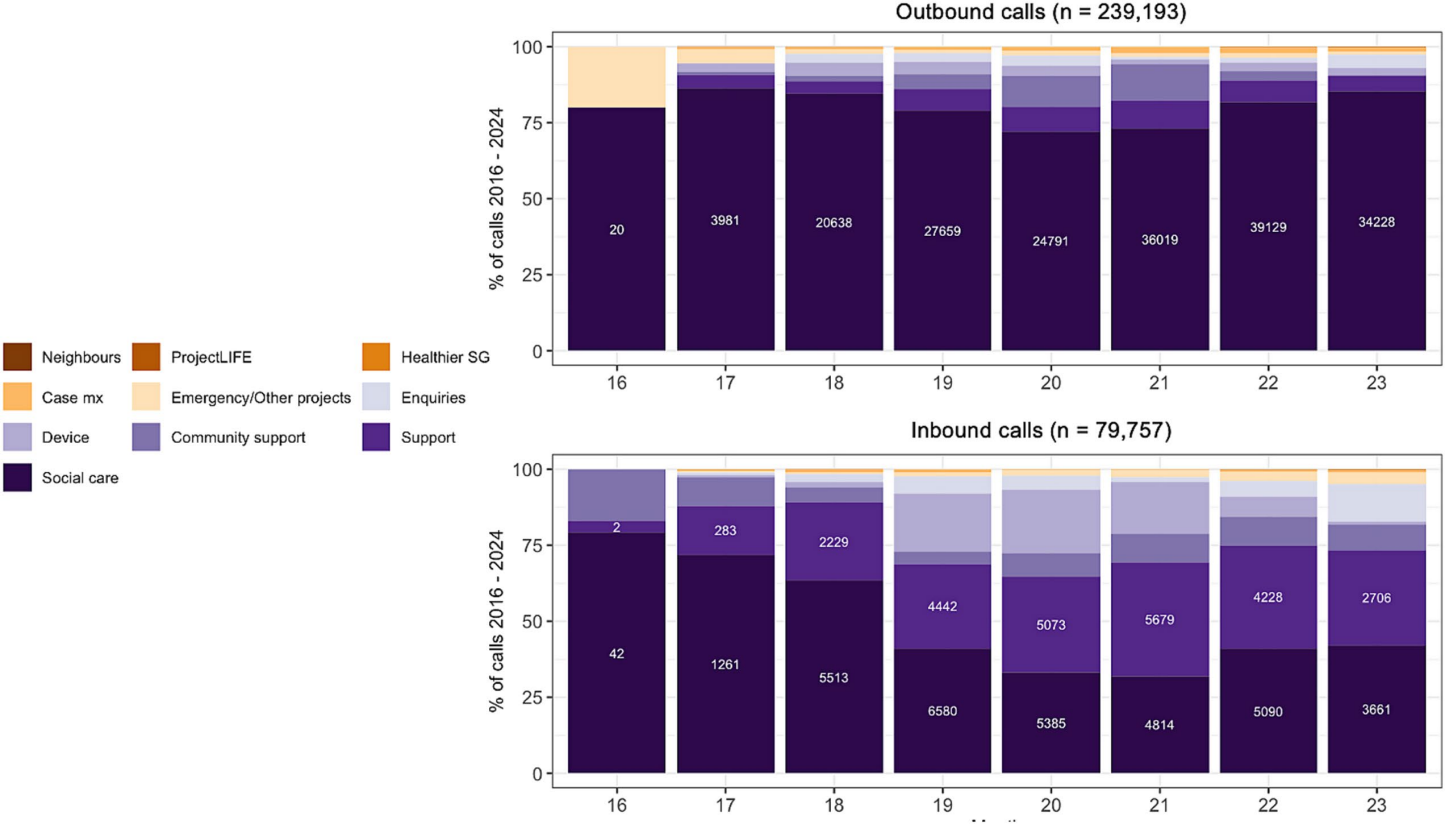
Inbound (initiated by CareLine users) and outbound (initiated by CareLine call agents) call volume.

There was an average of about 42,500 calls per year, with a total of 318,950 recorded calls over the time period. Call volume peaked at approximately 65,000 calls between 2020 and 2021, and constituted the majority (75.0%) of overall call volume. Both outbound and inbound calls were most commonly related to social care and emotional support, reflecting the program’s primary focus on providing social support and check-ins (Figure 2). And while most inbound calls were made during the day shift, there was a greater proportion of calls from those who lived alone during both the evening and midnight shifts.

The average number of outbound and inbound calls per user were 8.28 and 3.54, respectively, in 2017, and subsequently decreased to 4.46 and 0.93 in 2023. This was due in part to changes in the algorithm for call frequency to meet user needs, rapid recruitment rates, greater complexities in the nature of calls during the COVID-19 pandemic, as well as accompanying changes in workflow to prioritize case management and reduce excessive calls through partner collaborations.

Insufficient manpower for the team to manage rapid recruitment rates was also an issue that was highlighted by several CareLine staff during in-depth interviews. Participants highlighted several challenges, including the need for increased manpower especially during night shifts, and the recruitment of versatile staff who could speak different dialects and manage CareLine seniors.

*“There is a shortage of both call and recruitment teams as there is constant recruitment of new users. Manpower is also an issue because it’s challenging to recruit staff who…can speak different dialects, have similar prior experiences, and the learning curve is steep in terms of learning how to manage participants, especially difficult ones.”* (ID_001)

### User engagement

We also used the number of inbound calls made by users to estimate their engagement with the telecare service. Of the 9,512 unique users, we found that 19.7% (*n* = 1974) of the users never made an inbound call, 36.4% (*n* = 3,461) of the users made one inbound call, while 43.9% (*n* = 4,177) of the users made two or more inbound calls (median = 3) from the time of their recruitment. For those who made inbound calls, these calls were most commonly for social chats, emotional support, caring calls, and general service enquiries.

To further investigate if certain groups were more likely to make more inbound calls to CareLine than others, we conducted a multivariable regression to explore factors associated with making inbound calls overall, as well as those associated with making inbound calls having to do with either social chats or emotional support. We found that male users were more likely to make inbound calls overall (OR: 1.28; 95% CI: 1.26–1.30), as well as specifically for social chats and emotional support (OR: 1.48; 95% CI: 1.44–1.53) compared to females. Additionally, those who reported as not currently married were also more likely to make inbound calls; compared to those were not married, those who were widowed were almost five times more likely to make a call relating to social support or emotional support (OR: 4.48; 95% CI: 4.25–4.72). We also found that living arrangements were a predictor of inbound calls – those who lived alone were 1.52 times (95% CI: 1.49–1.55) more likely to make inbound calls overall, and 1.77 times (95% CI: 1.71–1.82) more likely to make calls related to social chats and emotional support (Table 2).

**Table 2:** Multivariable regression of factors associated with making inbound calls to CareLine.

Variable	Making inbound calls overall		Making inbound calls for social chats and emotional support	
	OR	95% CI	OR	95% CI
Gender (ref = Female)***				
Male	1.28	1.26–1.30	1.48	1.44–1.53
Ethnicity (ref = Chinese)***				
Malay	0.74	0.71–0.76	0.50	0.47–0.53
Indian	0.92	0.89–0.96	0.55	0.52–0.59
Other	0.78	0.72–0.84	0.49	0.42–0.58
Marital (ref = Married)***				
Divorced	1.14	1.10–1.18	1.36	1.26–1.46
Separated	1.34	1.23–1.45	1.26	1.05–1.49
Single	1.25	1.22–1.28	2.00	1.89–2.12
Widowed	1.46	1.42–1.50	4.48	4.25–4.72
Living (ref = Living w/others)***				
Living alone	1.52	1.49–1.55	1.77	1.71–1.82

In-depth interviews with 15 CareLine users also provided further insights into their perceptions of the program, as well as why they might make an inbound call to CareLine. Most participants expressed their appreciation for the connection they built over time with CareLine call agents, especially the ability to connect with them in languages (i.e., dialects) that they are comfortable with. One participant stated:

*“I really like calling agents just to chat…and I like [that] they speak to me in dialect or Chinese. It’s easier for me to understand. I used to call at night when I feel bored but now I call in the daytime…sometimes [the conversation] will last about 30 minutes. I call just to chat lah, but I can also ask questions about CPF or [new policies].”* (ID_003)

When asked about whether they feel any hesitance in calling, some participants stated that they have no reason to but they appreciate it when someone calls to check in on them. One participant also said:

*“At first I thought calling CareLine is like calling ‘999’ so I don’t want to disturb…in case other people need to call also.”* (ID_005)

### Complex callers and types

While call frequency data can provide some insights into user engagement, interpretation of this data is complex. A greater number of inbound calls may reflect high engagement, but could also suggest dependency or unresolved issues. Conversely, no calls might signal that users are well-supported, yet it could also indicate disengagement or barriers to accessing help. Hence, we looked at other call types and characteristics to further understand user needs and service effectiveness.

One such area is frequent callers. In our study, we defined frequent callers as those making 26 or more call a year, equivalent to a call every 2 weeks. Although they made up less than 1% (*n* = 64), frequent callers accounted for 68.5% of all inbound calls (*n* = 54,608). Of these, 23,057 calls (42.2%) of the calls were related to social care. Since the point of their enrolment, frequent callers made a median of 89.83 calls per year, with a maximum of 724.33 inbound calls per year. Frequent callers in CareLine also tended to be older with 79.7% of the group over the age of 70, and a higher proportion (54.7%) who lived alone (Table 1).

Another area of complexity was emergency calls. Emergency calls, though a smaller fraction of the total call volume, represent a critical aspect of CareLine’s services. On average, there were 1,156 inbound emergency calls recorded, with most of them (78.6%) having to do with calling for an ambulance. Emergency calls were made by 235 unique users, and almost all (89.8%) were enrolled in CareLine for less than a year before they made their first emergency call. Of the 235 users who made emergency calls, 24.2% of them were frequent callers. Further, those were made emergency calls tended to be older and over 75 years (61.9%), and not currently married (68.8%).

We also used qualitative feedback to further understand why users called CareLine for an ambulance instead of “995” for the nationwide emergency ambulance service. In particular, participants highlighted their appreciation for CareLine’s rapid response and 24/7 availability. They also liked the “*ease of communication with call agents who knew them*” and could converse in dialects, enhancing the sense of being understood and supported. One participant said also said they called CareLine to avoid unnecessary charges:

*“I would not be alive if not for CareLine…I called the when I fainted and they coordinated all the ambulance service for me. I call CareLine instead of the ambulance because I also do not want to anyhow call 995 and get charged for it if [my illness] is not serious enough for admission.”* (ID006).

## Discussion

The findings from our evaluation of the CareLine service offer significant insights into the complexities of telephone-based care interventions for older adults in Singapore and other similar contexts.

### User engagement and call characteristics

First, our study revealed marked gaps in user engagement, as indicated by the frequency and nature of inbound calls. This variation is consistent with findings from other studies on telephone-based services for older adults. For instance, a study by Cattan et al. (8) on telephone befriending services in the UK highlighted similar patterns, with a small group of users accounting for a disproportionately high number of calls, suggesting a high dependency on the service among this subgroup. Such frequent use may indicate significant unmet needs, whether emotional or practical, that users feel can be addressed through these services. This aligns with our finding that frequent callers were typically older and more likely to live alone, echoing previous research that identifies social isolation as a critical driver of engagement with befriending services (9).

Conversely, the substantial proportion of users who never made inbound calls or did so infrequently raises important questions about the accessibility and perceived utility of CareLine among these users. Similar observations have been made in studies evaluating the impact of telecare services, where low engagement sometimes correlates with a lack of awareness or understanding of the service’s full benefits (10). However, hypothetically, one would possibly argue the low engagement may be due to users’ better awareness of CareLine’s limitations and their capability to reach out for supports, particularly the younger senior cohort who are better educated and savvy in the know-how. This suggests that while CareLine is effectively reaching those in acute need, there is room to enhance outreach efforts, particularly to ensure that all users—especially those most vulnerable—are fully aware of and able to utilize the service’s resources, and to allocate resources strategically and make progressive service modification to also cater for the prospective needs of seniors who are well-informed and educated.

### Complex callers and types

The phenomenon of frequent users callers, identified in our study as those making 26 or more calls per year, is well-documented in the literature on telehealth and telephone-based services. Previous studies (16) have noted that frequent callers often have complex health and social care needs, which may not be fully addressed by standard service offerings. Our finding that frequent callers contributed to over two-thirds of all inbound calls is particularly significant, as it underscores the potential for these users to monopolize service resources, potentially at the expense of other.

The high volume of emergency calls routed through CareLine, particularly those related to ambulance services, also mirrors findings from other studies of telecare systems. Research by Pols (17) on telecare in the Netherlands found that users often prefer telecare services over direct emergency lines due to the perceived trustworthiness and personal connection with telecare staff. Our qualitative data support this, revealing that users value the familiarity and understanding provided by CareLine agents, who can communicate in preferred dialects and offer personalized support. The familiarity is also enabled by CareLine’s CRM and telephony integration. When a registered user calls, their profile instantly appears, allowing agents to recognize them and communicate in their preferred language. This also spares them from repeating personal details during distressing times. However, this reliance on CareLine for emergency situations also points to a potential gap in users’ understanding of when and how to use emergency services appropriately. Similar concerns were raised in a study (18) on the overuse of emergency services among telecare users in Ireland, which recommended targeted education for users to mitigate this issue.

### Language and cultural sensitivity

The importance of language and cultural sensitivity in the success of CareLine cannot be overstated. This finding is consistent with literature that emphasizes the critical role of culturally competent care in improving the effectiveness of health and social services for diverse populations. A study (19) culturally competent healthcare services in the United States found that services which incorporate patients’ language preferences and cultural context are more likely to be utilized effectively and produce positive outcomes. Our participants’ preference for communicating in their native dialects underscores the need for continued investment in recruiting and training staff who are not only linguistically capable but also culturally aware.

This finding is also supported by a study (20) which examined the importance of cultural sensitivity in elder care services for Chinese immigrants in Canada, finding that older adults are more likely to engage with services that they feel understand and respect their cultural background. This underscores the need for CareLine to maintain and possibly expand its capacity to provide services in multiple dialects, ensuring that it remains accessible and relevant to its diverse user base.

### Operational challenges

The operational challenges identified in our study, particularly regarding staffing and resource allocation, are common in similar services worldwide. For instance, a study (21) on the challenges faced by telephone-based support services in the UK found that insufficient staffing and high user demand were significant barriers to effective service delivery. The rapid increase in CareLine’s user base, especially during the COVID-19 pandemic, has exacerbated these challenges, highlighting the need for strategic resource management. To address these challenges, studies suggest the adoption of more efficient triage systems and the integration of technology to support service delivery. Similarly, our study points to the potential benefits of leveraging technology, such as automated call triaging or advanced case management systems, to alleviate some of the pressures on CareLine staff. Additionally, partnerships with other organizations could expand CareLine’s capacity, as suggested by a study on telecare services (22) found that collaboration with community health workers and volunteer groups significantly enhanced service delivery and user satisfaction.

### Implications for policy and practice

The insights from our evaluation of CareLine have important implications for the design and implementation of similar services, both within Singapore and globally. The need for a balanced approach that combines personalized care with efficient resource management is evident. This aligns with recommendations from a systematic review (23) on the effectiveness of befriending interventions, which emphasize the importance of scalability while maintaining personalized user experiences. Moreover, our findings underscore the importance of culturally sensitive approaches in elder care, particularly in multicultural societies. Services like CareLine must be flexible and adaptive, capable of meeting the diverse needs of their users. This requires ongoing investment in staff training and development, as well as regular feedback mechanisms to ensure that services remain responsive and effective. A study (24) on the adaptability of health services highlights the importance of continuous feedback and adaptation to meet the evolving needs of service users.

### Strengths, limitations, and future research

A key strength of this evaluation lies in its mixed-methods approach, which enabled us to capture both service utilization patterns and the lived experiences of users and providers. However, potential biases must be acknowledged. For instance, those who are most socially isolated may not enroll in CareLine at all, meaning that the service may not fully capture the most at-risk individuals. Similarly, findings from Singapore—a context with a strong community health system and unique cultural characteristics—may not be fully generalizable to other countries.

Future research should explore the cost-effectiveness and scalability of CareLine and similar interventions, particularly in resource-constrained settings. Integration with digital health tools, such as mobile apps or AI-driven triaging, may also warrant investigation as a means of complementing human-centered support. Longitudinal studies could further illuminate how engagement with tele-befriending services evolves over time and how such services can adapt to the changing needs of aging populations.

## Conclusion

In conclusion, CareLine plays a vital role in supporting older adults living alone in Singapore, offering both routine social support and critical emergency assistance. While the service is highly valued by its users, especially for its personalized and culturally sensitive approach, there are challenges that must be addressed to ensure its sustainability and effectiveness. By focusing on targeted user engagement strategies, enhancing staff capacity, and leveraging technology, CareLine can continue to provide essential support to one of the most vulnerable segments of the population. The lessons learned from this evaluation can inform the development of similar interventions in other settings, contributing to the global effort to improve the quality of life for older adults through innovative and responsive care models.

## Data Availability

The raw data supporting the conclusions of this article will be made available by the authors, upon reasonable request.
