# Supplementary material for: Experiences, perceptions and utilization of CareLine: an evaluation study
Source: Front Public Health. 2025 Dec 11;13:1667535. doi: 10.3389/fpubh.2025.1667535 (PMC12738338; doi:10.3389/fpubh.2025.1667535)
Supplement: Supplementary file 1 [file Data_Sheet_1.docx]

Topic guide for CareLine users

| **Domain** | **Questions** |
| --- | --- |
| Introduction | - Do you currently live alone? - How would you describe your general health? - Could you tell me how you first got involved with CareLine? |
| Experiences with CareLine | - What has your experience of CareLine been so far? - What do you like most about this programme? - What do you think could be improved about the programme? - What do you generally use CareLine for? - Can you tell me about a specific CareLine experience you had? - How do you think CareLine has improved your life? |
| Alternate interventions | - Other than CareLine, do you participate in other programmes? |
| Loneliness | - Do you ever feel lonely?/Can you tell me what the word lonely means to you? - How would you describe the feelings? How does it make you feel? - (if participant says no) Why do you think others may feel lonely? - Would you say you feel lonely in your life at the moment? Are there situations or times when you feel more lonely? - Are there things that trigger your feelings of loneliness? - When you feel lonely, what do you do to feel better etc.? |

Topic guide for CareLine staff

| **Domain** | **Possible questions** |
| --- | --- |
| Introduction | - How long have you been working with CareLine? - In what capacity do you work with CareLine? - How did you first come to hear about/work with CareLine? |
| Benefits of CareLine | - How has your experience been working with CareLine participants? - Can you tell me through about an experience you’ve had with a CareLine participant? - What do you think works well with CareLine? - What do you think participants enjoy most about the programme? |
| Challenges with CareLine | - What do you think are some challenges with running CareLine? (i.e. staff levels, funding, logistics) - Are there any areas of the intervention that you think would be hard to keep providing? - Are there any other challenges that we have not mentioned? - What do you think participants find most challenging about the programme? |
